# Molecular differences between stromal cell populations from deciduous and permanent human teeth

**DOI:** 10.1186/s13287-015-0056-7

**Published:** 2015-04-18

**Authors:** Nina Kaukua, Mo Chen, Paolo Guarnieri, Markus Dahl, Mei Ling Lim, Tülay Yucel-Lindberg, Erik Sundström, Igor Adameyko, Jeremy J Mao, Kaj Fried

**Affiliations:** Center for Craniofacial Regeneration (CCR), Columbia University Medical Center, 630 W. 168 St. – PH7E, New York, NY 10032 USA; Department of Neuroscience, Karolinska Institutet, Stockholm, SE-171 77 Sweden; Department of Systems Biology, Herbert Irving Comprehensive Cancer Center, Columbia University Medical Center, New York, NY 10032 USA; Department of Dental Medicine, Karolinska Institutet, Huddinge, SE 14104 Sweden; Advanced Center for Translational Regenerative Medicine (ACTREM), Department for Clinical Science, Intervention and Technology (CLINTEC), Division of Ear, Nose and Throat, Karolinska Institutet, Huddinge, SE 141 86 Sweden; Division of Neurodegeneration, Department of Neurobiology, Care Sciences and Society, Karolinska Institutet, Huddinge, SE-14157 Sweden; Department of Physiology and Pharmacology, Karolinska Institutet, Stockholm, SE 171 77 Sweden

## Abstract

**Introduction:**

Deciduous and permanent human teeth represent an excellent model system to study aging of stromal populations. Aging is tightly connected to self-renewal and proliferation and thus, mapping potential molecular differences in these characteristics between populations constitutes an important task.

**Methods:**

Using specifically designed microarray panels, Real-Time Quantitative Polymerase Chain Reaction (RT q-PCR), Western blot, immunohistochemistry and siRNA-mediated knock down experiments, we have detected a number of molecules that were differentially expressed in dental pulp from deciduous and permanent teeth extracted from young children and adults, respectively.

**Results:**

Among the differentially regulated genes, high-mobility group AT-hook 2 (HMGA2), a stem cell-associated marker, stood out as a remarkable example with a robust expression in deciduous pulp cells. siRNA-mediated knock down of *HMGA2* expression in cultured deciduous pulp cells caused a down-regulated expression of the pluripotency marker *NANOG*. This finding indicates that *HMGA2* is a pulpal stem cell regulatory factor. In addition to this, we discovered that several proliferation-related genes, including *CDC2A* and *CDK4*, were up-regulated in deciduous pulp cells, while matrix genes *COL1A1*, fibronectin and several signaling molecules, such as *VEGF*, *FGFr-1* and *IGFr-1* were up-regulated in the pulp cells from permanent teeth.

**Conclusions:**

Taken together, our data suggest that deciduous pulp cells are more robust in self- renewal and proliferation, whereas adult dental pulp cells are more capable of signaling and matrix synthesis.

**Electronic supplementary material:**

The online version of this article (doi:10.1186/s13287-015-0056-7) contains supplementary material, which is available to authorized users.

## Introduction

Senescence and aging are associated with the loss of self-renewing capacity of stem cells. This principle is valid for multiple locations in the body, including the nervous system, connective tissue and bone marrow, and plays a significant role in the regenerative potential of stem cells [1-3]. There are several extrinsic and intrinsic elements that contribute to the aging of stem cells. These include changes in the systemic environment through factors from the blood or modifications of the stem cell niche, and alterations of elements within the stem cells such as protein accumulation, damage to mitochondrial as well as nuclear DNA, telomere attrition and cell cycle inhibition that eventually leads to failure of function [2,4]. Identification of potent tissue-specific stem cells and their banking is crucial for regenerative medicine. Teeth host pulpal stromal cells with subpopulations that have mesenchymal stem cell characteristics [5-7] that are easily extracted and amenable for manipulation. In animal models, these pulp cells seem to have a beneficial effect on spinal cord regeneration [8] and other types of trauma and disease, although their precise function is not clear and there is no evidence of any transdifferentiation into other cell types [9,10]. For reasons that remain elusive and contradictory to their apparent *in vivo* quiescence [11], dental pulp cells undergo rapid proliferation in *ex vivo* culture, apparently more rapid than bone marrow mesenchymal stem cells (BMMSCs) [5,12]. Previous work on genetic profiles of dental pulp cells has yielded several important clues [13-17]. Comparisons of gene expression between fast growing and slow growing pulp cell populations showed robust expression of transcription factors, *E2F2, PTTG1, TWIST-1*, and transcriptional cofactor, *LDB2*, each with critical roles in cell growth and survival, in dental pulp cells, just as in the periodontal ligament and bone marrow [12].

Here, we address the issue of molecular differences between pulp cells from deciduous and permanent teeth to elucidate the mechanism that provides high stemness and self-renewal capacity. These two types of teeth offer an excellent model for studies of molecular differences between stromal cell populations, especially in relation to their age. We approached this task by comparing genetic profiles from cells isolated from different origins and generations of human teeth with subsequent functional studies. We found that the genetic profile of deciduous dental pulp cells differs from that of adult dental pulp cells, due to ontogenic discontinuity between deciduous and permanent teeth.

## Methods

### Ethics statement

Deciduous teeth and permanent teeth extracted for medical-dental reasons after thorough diagnoses by clinicians were collected from patients following approval by The Institutional Review Board at the Columbia University Medical Center and a Regional Ethical Committee in Stockholm. Patients provided informed consent to participate in this study. When deciduous teeth were obtained from children, informed consent was given from the next of kin, caretakers, or guardians on behalf of the children enrolled in our study. Informed consent was recorded, with age and gender of the donor and parent/patient approval but without any personal identifiers.

The Regional Ethical Committee in Stockholm approved the isolation and establishment of human embryonic stem cell lines. The HS181 cell line used in this study was derived at Karolinska Institutet, Karolinska University Hospital, Huddinge, Sweden.

The procedures for the use of human tissues, adult and embryonic gingiva, for experimental studies were approved by the Regional Ethical Committee, Stockholm.

### Cell isolation

The deciduous teeth were collected from 3- to 12-year-old children (n = 8), and permanent teeth were collected from adults, 19 to 52 years of age (n = 8). Both deciduous and permanent teeth had full length roots with no visible root resorption and were extracted due to pericoronitis/periodontal issues, caries or orthodontic reasons. Cell isolation followed our previously described methods and approaches [9,18]. The tooth was mechanically separated into two halves to remove all soft tissue in the pulp chamber and the root canal. The pulp tissue was digested with 3 mg/ml collagenase I and 4 mg/ml dispase (Invitrogen, Carlsbad, CA, USA) for one hour at 37°C. The dissociated cells were passed through a 70-μm-cell strainer (BD Biosciences, San Jose, CA, USA) and plated in MEM-alpha medium (Invitrogen) containing 10% fetal bovine serum (Gibco, Grand Island, NY, USA) and penicillin-streptomycin (Atlanta Biologicals, Lawrenceville, GA, USA). The cells from each pulp were cultured separately and then passaged at 90% confluency in different 10 cm dishes (Nunc, Rochester, NY, USA) or several wells of the six-well plate model (BD Biosciences) for expansion, and utilized for further analysis. The analyses were performed on pulp cells from different individual pulps without pooling.

### siRNA transfection

Deciduous pulp cells were transfected with Dharmafect transfection reagent nr.4 (Thermo Scientific, Lafayette, CO, USA) according to the manufacturer’s protocol, with a final concentration of 100 nM siRNA ON-TARGETplus Human *HMGA2* and 100 nM siRNA ON-TARGETplus Human Control Pool (Thermo Scientific), for 48 hours. The cells were transfected after one passage, at 40% confluency in 10% fetal bovine serum MEM-alpha medium containing no antibiotics.

### RNA and protein extraction

RNA and protein were extracted from passage one cells with the Qiagen RNeasy Mini Kit (Valencia, CA, USA). At 90% confluency, cells were washed with PBS (Lonza, Walkersville, MD, USA) and then placed in lysis buffer following the manufacture’s protocols. For siRNA transfection experiments, total RNA was extracted according to the Qiagen AllPrep DNA/RNA/Protein Mini Kit protocol with a Qiagen AllPrep DNA/RNA/ Protein Mini Kit (Qiagen, Hilden, Germany).

### Microarray gene expression analysis

Samples were analyzed using a PIQOR Stem Cell Microarray chip (Miltenyi Biotec, Auburn, CA, USA). This consisted of 942 relevant genes for stem/progenitor cells and key genes for cell differentiation to identify gene sets that are differentially expressed between dental pulp cells in the deciduous and adult teeth. All microarray data have been deposited in a public repository, Gene Expression Omnibus (GEO), with accession number GSE58668.

The isolated RNA was subjected to microarray analyses. A total of 1 μg RNA for each sample was used for amplification and further analysis with the PIQOR stem cell microarray chip, followed by detection with a laser scanner (Agilent Technologies, Santa Clara, CA, USA). The dataset consisted of two microarrays, each containing 11 slides for a total of 16 samples. The Linear Model for Microarray Data package (Limma), within R/Bioconductor statistical framework, was used to pre-process the raw signal intensities, perform quality controls and estimate statistical significance [19]. Expression intensities were background-corrected using a convolution of normal and exponential distributions with an offset of 50, which were then normalized within every slide using LOESS normalization. We analyzed the red and green channels separately using the mixed model method and different groups were quantile-normalized separately. The correlation was computed between the two channels for the same spot. Differentially expressed genes for the eight different comparisons considered were identified by computing the contrasts, fitting to a linear model, performing the hypothesis tests and correcting for multiple testing using the Benjamin-Hochberg correction (p ≤ 0.05). DAVID [20,21] was used to test over-represented functional groups, and the output was further analyzed with Enrichment Map [22] a plugin for Cytoscape [23]. Additionally we used Ingenuity Pathway Analysis (IPA)(Ingenuity®Systems) to generate the network of connections between modulated genes, using the knowledge-based topology. We then isolated the *HMGA2* to carry out the causal network analysis with IPA.

### cDNA synthesis and real-time quantitative RT q-PCR

RNA was isolated with the Qiagen RNeasy Mini Kit (Qiagen). DNA contamination was eliminated with DNase treatment (Applied Biosystems, Foster City, CA, USA). cDNA was made according to, and with supplies from, Applied Biosystems. A real-time quantitative PCR procedure was performed with cDNA, Taqman Universal PCR mastermix and gene expression assays (Table 1) using the Applied Biosystems ViiA 7™ Real-Time PCR System. Data were analyzed with the relative comparative method, where *GAPDH* was used to normalize the C_t_ value. Deciduous dental pulp cells were set as control to calculate the differences from the permanent dental pulp cells.

**Table 1:** **Primers for RT q-PCR**

**Gene symbol**	**Assay**	**Gene symbol**	**Assay**
*GAPDH*	Hs99999905_m1	*VEGF*	Hs00900054_m1
*HMGA2*	Hs00171569_m1	*FGFR1*	Hs00241111_m1
*FABE*	Hs02339439_g1	*PLXNA3*	Hs00250178_m1
*CDK1*	Hs00364293_m1	*TIMP1*	Hs99999139_m1
*MAD2L1*	Hs03063324_g1	*SERPINF1*	Hs01106937_m1
*MMP6*	Hs00757922_g1	*JMJ (Jarid2)*	Hs01004457_m1
*CDK4*	Hs00364847_m1	*IGF1R*	Hs00951562_m1
*MMP23A;B*	Hs00270380_m1	*COL1A1*	Hs00164004_m1
*TK1*	Hs01062125_m1	*INHBA*	Hs00170103_m1
*ITGB1*	Hs00559595_m1	*FN1*	Hs00277509_m1

For siRNA transfection experiments, cDNA synthesis was performed using the High Capacity cDNA Reverse Transcription Kit (Invitrogen, Foster City, USA) protocol. Further on, the RT q-PCR procedure was performed according to an Applied Biosystems protocol with gene expression assays *GAPDH* (FAM, Hs02758991_g1), *NANOG* (FAM, Hs02387400_g1), *OCT4* (Hs03005111_g1) and *HMGA2* (FAM, Hs00971725_m1) (Applied Biosystems, Foster City, CA, USA). PCR reactions were applied on ABI 7500 Fast- Real Time PCR System. As above, the relative comparative method was used to analyze data.

The cells from the HS181 cell line were maintained in knockout Dulbecco’s modified Eagle’s medium supplemented with 20% knockout serum replacement, 2 mM glutamax, 0.5% penicillin–streptomycin, 1% non-essential amino acids, 0.5 mM 2-mercaptoethanol and 8 ng/ml basic fibroblast growth factor (bFGF) (all from Life Technologies, Invitrogen, Stockholm, Sweden).

BMMSCs (Life Technologies, Invitrogen) at passage 3 were cultured in MesenPRO RS basal medium, supplemented with low serum of 2% MesenPRO RS growth supplement and 1% penicillin and streptomycin (Life Technologies, Invitrogen).

Total RNA was purified according to the manufacturer’s instructions from deciduous pulp cells, HS181 and BMMSCs using a commercially available RNeasy Mini Kit (Qiagen, Germany). cDNA samples were prepared from 100 ng of total RNA with superscript III (Invitrogen) using a Veriti thermal cycler (Applied Biosystems, Stockholm, Sweden). RT q-PCR was processed on a *7500* Fast Real-Time PCR System (Applied Biosystems, USA) with Taqman Universal Master Mix and Taqman primer/probe for *OCT4* (Hs03005111_g1), *NANOG* (Hs04260366_g1) and *HMGA2* (Hs00971725_m1). The housekeeping gene, *GAPDH* (Hs02758991_g1) was used as an endogenous control. The expression level for each sample was normalized to *GAPDH* and quantification of expression was estimated using the relative comparative method and was presented as relative fold change.

### SDS-PAGE and Western blot

Proteins were isolated with the Qiagen RNeasy Mini Kit followed by acetone precipitation (deciduous pulps: n = 3, permanent pulps: n = 3). The protein extract was dissolved in 1% SDS. Protein concentration was measured with a Biorad RC DC Protein Assay Kit and SmartSpec Plus Spectrophotometer. A total of 20 μg of protein was loaded into wells on NuPAGE® Novex 4% to 12% Bis-Tris Gels and then transferred onto polyvinylidene difluoride (PVDF) membranes (Biorad, Hercules, CA, USA) using electroblotting. The membranes were blocked with blocking buffer (Licor, Lincoln, NE, USA) and then probed with the antibodies for beta-actin (1:500, Santa Cruz Biotechnology, Santa Cruz, CA, USA), cdc2 (1:2000, Cell Signaling Technology, Danvers, MA, USA), Fatty Acid Binding Protein 5 (1 μg/ml, Abcam, Cambridge, MA, USA), HMGA2 (1:1000, Cell Signaling Technology) and PEDF (aka Serpinf1, 2 μg/ml, Abcam) overnight in 4°C on a shaker. Thereafter, the corresponding secondary antibodies goat anti-mouse immunoglobulin G (IgG)-AP and goat anti-rabbit IgG-AP (Santa Cruz Biotechnology) were added for one hour at room temperature on a shaker. Proteins were detected using the 1-Step NBT/BC1P system (Thermo Scientific, Rockford, IL, USA). Quantification of the intensity levels of the Western blot bands was performed with ImageJ software.

### Immunohistochemistry

Deciduous teeth were collected from 3- to 11-year-old patients (n = 3) and permanent teeth were collected from adult patients (19 to 81 years of age, n = 3). Teeth were cracked open and pulps were collected and snap frozen in optimal cutting temperature (OCT) cryomount (Histolab, Gothenburg, Sweden) for subsequent 5 μm sectioning. The sections were fixed for 20 minutes in 4% paraformaldehyde (Merck, Darmstadt, Germany), rinsed and then incubated overnight at room temperature with primary antibodies against HMGA2 (1:500, Cell Signaling Technology) and vimentin (1:200, Abcam). Additional staining was performed the following day with 4',6-diamidino-2-phenylindole (DAPI) (Invitrogen, Burlington, Canada) at 300 nM applied for three minutes. Secondary antibodies used were Alexa Fluor® 555 Donkey Anti-Rabbit IgG (H + L) and Alexa Fluor® 488 Goat Anti-Chicken IgG (H + L) (Invitrogen, Eugene, OR, USA). Control sections were incubated where the primary antibody was omitted. This resulted in no detectable artifact. Additional negative control stainings were performed with isotype control for rabbit primary antibody (Invitrogen, Eugene, OR, USA), which resulted in no unspecific staining. Confocal microscopy was performed with Zeiss LSM700 CLSM and image processing was carried out with Imaris software.

### Hematoxylin & eosin staining of deciduous and permanent tooth pulp

Slides with pulp sections were washed in distilled water for one minute. They were then immersed in modified Mayers solution hematoxylin (Histolab, Gothenburg, Sweden) for ten minutes, followed by rinsing in distilled water. Subsequently, slides were incubated in 0.2% eosin (Histolab) rinsed, dehydrated in graded steps of ethanol and xylene (VWR Chemicals, Fontenay-sous-Bois, France) and then mounted in Entellan (Merck). Light microscopy was performed with a Nikon Eclipse E600 microscope and images were processed in Adobe Photoshop CS5.

### Immunohistochemistry of embryonic and adult gingiva

Human fetal lower jaw tissue (8.5 weeks after conception) was retrieved from elective routine abortions, with written consent from the pregnant women. The specimens (n = 2) were immersed in 4% paraformaldehyde (Merck) overnight and then rinsed in 30% sucrose for 24 hours. They were then snap frozen in OCT cryomount (Histolab) for subsequent sectioning. Biopsies of human healthy gingiva from patients 54- to 67-years old (n = 3) were obtained with written consent from the donors, after approval by the Regional Ethical Committee, Stockholm. These samples were fixed in paraformaldehyde, rinsed, dehydrated and embedded in paraffin according to standard methods. After sectioning and deparaffination, they were processed for immunohistochemistry. Immunohistochemical staining was performed as described above with HMGA2 antibody (1:500, Cell Signaling Technology), CD31 antibody (1:300, Dako, Glostrup, Denmark) and Vectashield mounting medium with DAPI (Vector Laboratories, Burlingame, CA, USA). Imaging was performed using a confocal microscope, Zeiss LSM710 CLSM, and image processing was carried out with Imaris software.

## Results

### Microarray gene expression analysis reveals differences between deciduous and permanent pulp cells

**Figure 1:**
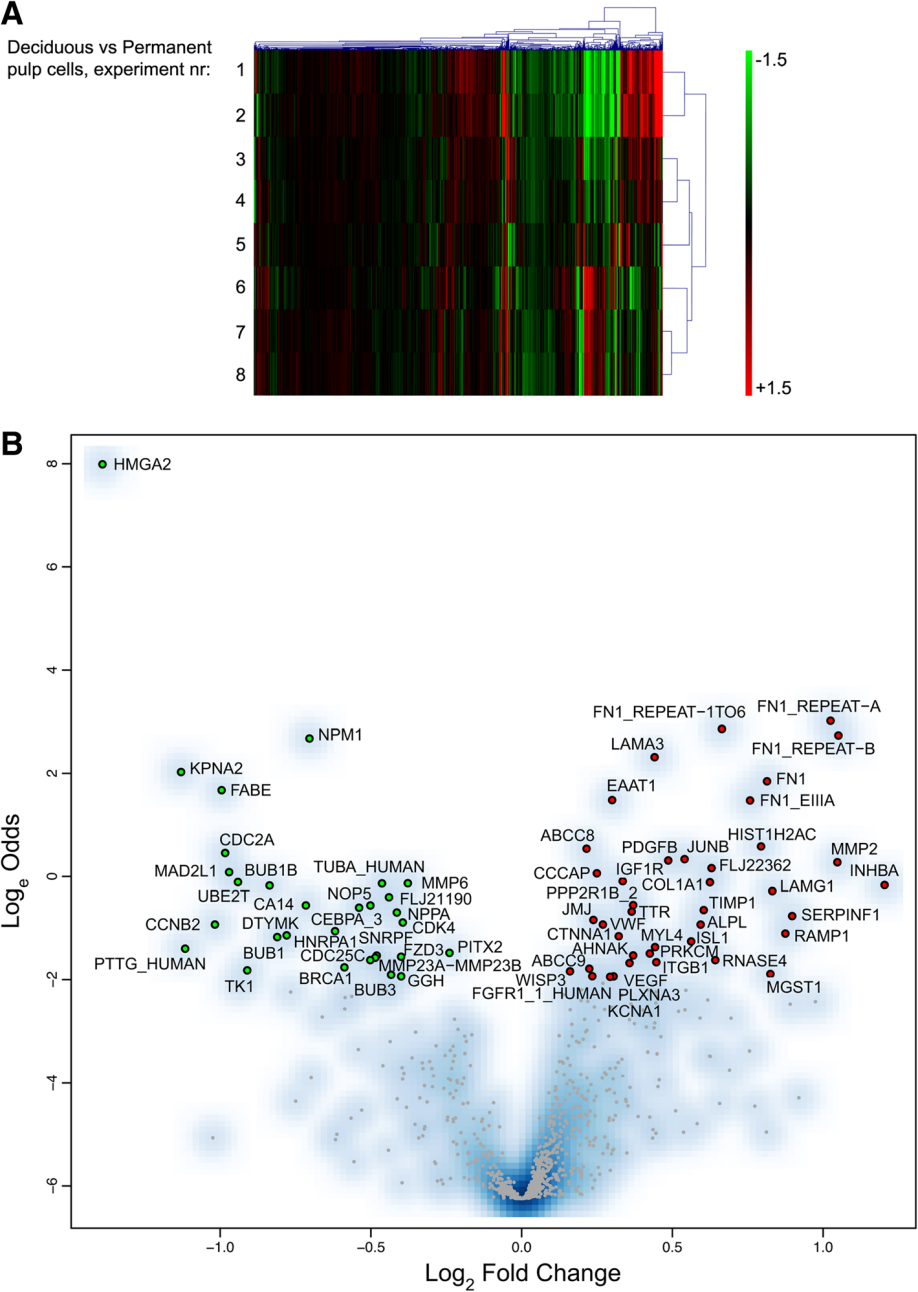
Heat map and volcano plot of genes in permanent versus deciduous pulp cells. **A)** Heat map of gene expression level in permanent pulp cells versus deciduous pulp cells. Each panel illustrates one comparison between permanent pulp cells and deciduous pulp cells from different subjects (n = 8). Red color indicates a high gene expression level, while green color means a low expression level in permanent compared to deciduous pulp cells. The data are z-score transformed. **B)** Volcano plot of up- and down-regulated genes in permanent versus deciduous pulp cells. Listed up- or down-regulated gene names have a significance of *P* ≤ .05.

In order to tackle age differences in populations of connective tissue cells, we extracted cells from deciduous and permanent teeth obtained from young children and adults. Next we analyzed the transcriptomes from these cells using custom-generated microarray to discover differentially expressed transcripts related to stemness, proliferation and extracellular matrix production. We discovered genes that were differentially expressed by cells from deciduous versus adult teeth, as identified by cross-comparative analysis. Differences in hybridization intensities were observed for a proportion of the expressed genes as shown in the heat map (Figure 1A). The expression of a total set of 70 genes was significantly different (*P* ≤0.05, Additional file 1) and is shown in Figure 1B. Comparing deciduous to permanent teeth, 40 genes were up-regulated, and 30 genes were down-regulated. Genes associated with cell division, extracellular matrix components, differentiation and aging differed remarkably between the deciduous and permanent pulp cells (Tables 2 and 3).

**Table 2:** **Up-regulated genes in pulp cells from permanent teeth in microarray**

**Gene name**	**Reference sequence**	**Function**	**Log**_**2**_ **fold change**
*INHBA*	NM_002192	Regulation of nerve cell survival, embryonic axial development and bone growth	1.204
*SERPINF1*	NM_002615	Inhibits angiogenesis, is a neurotrophic factor involved in neuronal differentiation in retinoblastoma cells	0.897
*FN1*	NM_002026,NM_212474, NM_212475, NM_212476,NM_212478, NM_212482	Cell adhesion, motility, opsonization, wound healing, and maintenance of cell shape	0.814
*COL1A1*	NM_000088	Found in most connective tissues	0.625
*TIMP1*	NM_003254	Inhibitors of the matrix metalloproteinases, which are involved in degradation of the extracellular matrix. They promote cell proliferation in a wide range of cell types.	0.604
*ITGB1*	NM_002211,NM_033666, NM_033667,NM_033668, NM_033669,NM_133376	Receptors for collagen, fibronectin, fibrinogen and laminin	0.447
*VEGF*	NM_001025366,NM_001025367,NM_001025368, NM_001025369	Active in angiogenesis, vasculogenesis and endothelial cell growth	0.358
*IGF1R*	NM_000875	Binds insulin-like growth factor	0.335
*PLXNA3*	NM_017514	Epithelial and neural tissue development	0.293
*FGFR1_1_HUMAN*	NM_015850,NM_023105, NM_023106,NM_023107 NM_023108,NM_023110	Interacts with fibroblast growth factors, influences mitogenesis and differentiation	0.234
*JMJ (Jarid2)*	NM_004973	Stem cell differentiation, negative regulation of cell proliferation	0.238

**Table 3:** **Down-regulated genes in pulp cells from permanent teeth in microarray**

**Gene name**	**Reference sequence**	**Function**	**Log**_**2**_ **fold change**
*HMGA2*	NM_003483	Expressed during mammalian embryonic development. High expression in neural stem cells and decreases with age	−1.390
*FABE*	NM_001444	Found in epidermal cells, may be involved in keratinocyte differentiation	−0.995
*CDC2A*	NM_001786, NM_033379	Cell cycle key mediator of neuronal cell death in brain development and degeneration	−0.983
*MAD2L1*	NM_002358	Cell division and mitosis	−0.970
*TK1*	NM_003258	Cell division and mitosis	−0.910
*MMP23A-MMP23B*	NM_006983, NR_002946	Cytosolic enzyme, high in proliferating cells	−0.486
*FZD3*	NM_017412	Involved in the breakdown of extracellular matrix	−0.400
*MMP6*	NM_005792	Cell division	−0.378
*CDK4*	NM_000075	Cell cycle	−0.394
*PTTG_human*	NM_004219,NM_006607, NR_002734	Cell division	−1.116

Genes encoding extracellular matrix proteins, including collagen type I *(COL1A1)* and fibronectin were differentially expressed in favor of pulp cells from permanent teeth. In addition, genes associated with cell differentiation were robustly expressed by pulp cells of the permanent teeth, including *INHBA, SERPINF1, PLXNA3* and *JMJ*. In comparison, genes promoting cell proliferation, mitosis and division, including *MAD2L1, MMP23A-MMP23B, MMP6* and *PTTG*, were highly expressed in pulp cells from deciduous teeth. Remarkably, *HMGA2*, a gene strongly expressed by neural stem cells during embryogenesis, was highly expressed in pulp cells of deciduous but not permanent teeth (Figure 1B and Table 3). The differences in mRNA expression are significant with the moderated T-test, *P* ≤0.05.

IPA revealed that several genes are in association with *HMGA2*, in particular genes that are involved with stemness, tumorigenesis and cell regulation, such as *SP1, BRCA1* and *cyclin A* (Additional files 2 and 3).

**Figure 2:**
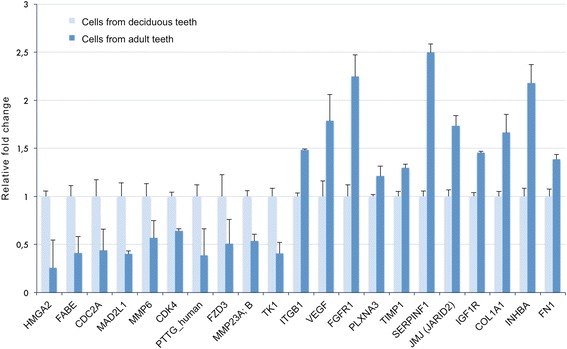
RT q-PCR of a subset of genes from the microarray. Results from RT q-PCR analysis of a subset of up- or down-regulated genes in permanent pulp cells versus deciduous pulp cells. The data agree with the results obtained in the microarray experiments, and were all significantly different, *P* ≤0.05, Student’s t-test.

**Figure 3:**
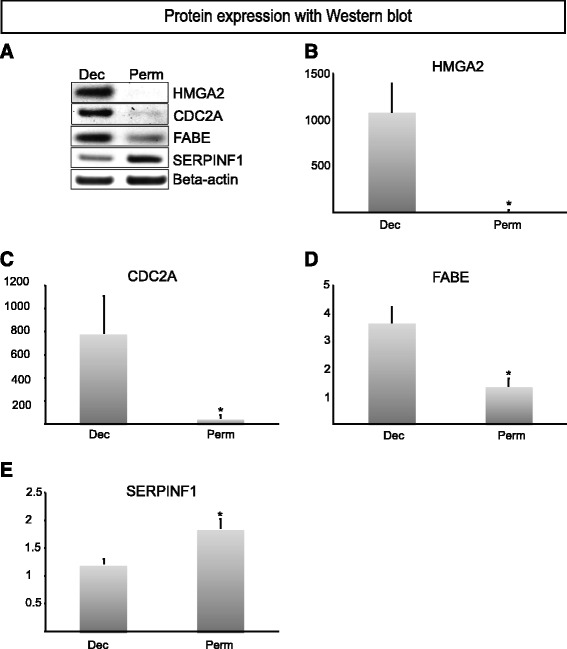
Western blot analysis of protein expression. Western blot analysis of proteins reflects the results observed in the microarray and RT q-PCR gene experiments. Note that HMGA2 protein is low or non-existent in permanent pulp cells. Quantification on the intensity levels of the Western blot bands was performed, B-E. **A)** Western blot bands, **B)** HMGA2, **C)** CDC2A, **D)** FABE, **E)** SERPINF1. **Dec**: Pulp cells from deciduous teeth, n = 3 **Perm**: Pulp cells from permanent teeth, n = 3. *indicates *P* ≤ 0.05, unpaired Student’s t-test.

To validate the microarray analysis, RT q-PCR was performed for genes of interest, especially those involved in cell proliferation and mitosis (Tables 2 and 3). This confirmed the microarray data obtained (Figure 2) with significant differences (*P* ≤0.05). For example, genes that are involved in cell cycle division and mitosis, such as *TK1*, *CDK4* and *MMP6*, were highly expressed in cells from deciduous teeth. We further probed the expression of proteins coded by selected genes that were detected by microarray and RT q-PCR. Western blot analysis and quantification demonstrated that HMGA2, FABE and CDC2A protein levels were elevated in cells from deciduous teeth while SERPINF1 had a higher expression in pulp cells from permanent teeth (Figure 3A-E).

### siRNA-mediated loss-of-function experiments show association between HMGA2 and NANOG in pulp cells

**Figure 4:**
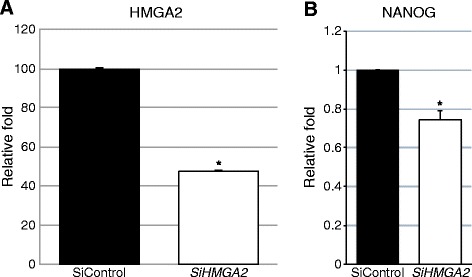
*HMGA2* siRNA transfection of deciduous pulp cells *in vitro*. **A)** There is an endogenous reduction of *HMGA2* mRNA levels compared to control conditions, *P* ≤0.05. **B)** Endogenous reduction of *HMGA2* mRNA levels reduces the expression of *NANOG* as compared to control conditions. *indicates *P* ≤0.05, Student’s t-test.

To elucidate the importance of elevated levels of *HMGA2* in deciduous teeth, we suppressed the *HMGA2* levels by siRNA transfections in deciduous pulp cells and compared the expression of a stem cell marker in siControl (untreated samples) versus *siHMGA2* treatments (Figure 4A). The results demonstrated that at 48 hours after transfection the expression of the stem cell gene *NANOG* was reduced (Figure 4B), although *SOX2* and *OCT4* expressions were unchanged. The graph shows standard error of the mean, normalized to the housekeeping gene GAPDH. The difference in mRNA expression between siControl versus *siHMGA2* is significant with the Student’s t-test, *P* ≤0.05.

**Figure 5:**
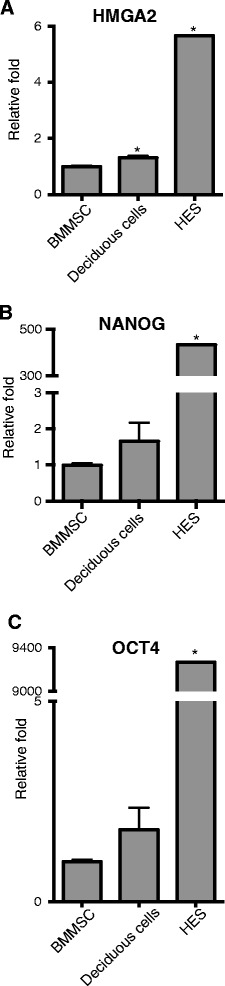
RT q-PCR of BMMSCs, deciduous pulp cells and HES. BMMSCs was set as the standard that deciduous pulp cells and HES were compared to with relative fold change. **A)** *HMGA2* gene expression, **B)** *NANOG* gene expression, **C)** OCT4 gene expression. *indicates *P* ≤0.05, Student’s t-test. BMMSCs: bone marrow mesenchymal stem cells; HES**:** human embryonic stem cells.

For comparisons of pluripotency markers, RT q-PCR levels of *HMGA2*, *NANOG* and *OCT4* were determined simultaneously in decidous pulp cells, BMMSCs and human embryonic stem cells (HES). As expected, HES displayed very high levels of HMGA2, *NANOG* and *OCT4* expression compared to deciduous pulp cells and BMMSCs. However, deciduous pulp cells and BMMSCs had similar expression levels with regard to these markers (Figure 5).

### Immunohistochemistry reveals that HMGA2 expression in deciduous pulp tissue is unrelated to blood vessels

**Figure 6:**
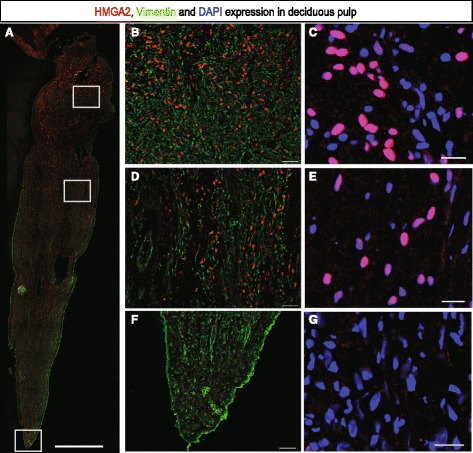
HMGA2 expression in deciduous pulp. HMGA2 is abundantly expressed in deciduous pulp but only by a subpopulation of cells. **A)** HMGA2 (red), vimentin (green). **B-G** are zoomed in areas. C, E, G show co-localization of DAPI staining and HMGA2. A: scale bar 1,000 μm; B, D and F: 50 μm; C, E and G: scale bar 20 μm. DAPI, 4',6-diamidino-2-phenylindole.

**Figure 7:**
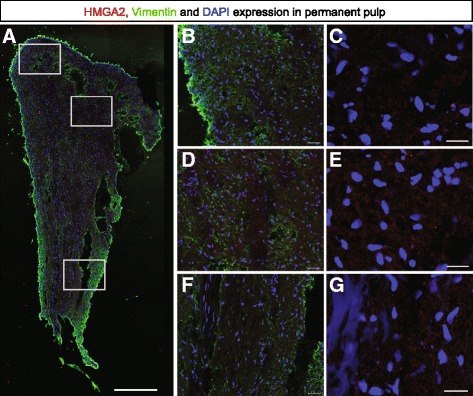
HMGA2 expression in permanent pulp. HMGA2 expression is low or non-existent in permanent pulp. **A)** HMGA2 (red), vimentin (green) and DAPI (blue). **B-G** are zoomed in areas. A: scale bar 500 μm; B, D, F: scale bar 40 μm; C, E and G: scale bar 20 μm. DAPI, 4',6-diamidino-2-phenylindole.

**Figure 8:**
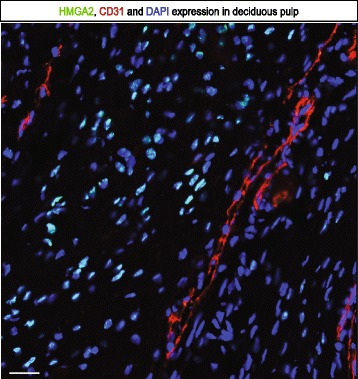
Immunohistochemistry of HMGA2 expression and blood vessels in deciduous pulp. HMGA2 does not co-localize with blood vessels in deciduous tooth pulp, HMGA2 (green), CD31 (red) and DAPI (blue). Scale bar 30 μm. DAPI, 4',6-diamidino-2-phenylindole.

Sections from deciduous tooth pulps showed a marked nuclear HMGA2 immunoreactivity in stromal cells, while no corresponding immunostaining was observed in permanent pulp tissue (Figure 6A-G and Figure 7A-G). HMGA2 immunoreactivity was not omnipresent in deciduous pulps, but rather seemed to be selectively expressed in a subpopulation of cells (Figure 6C, E and G). The proportion of HMGA2-positive cells was quantified and found to be 16.86 ± 2.28% (Additional file 4). The organization of deciduous and permanent tooth pulps, respectively, is displayed in Additional file 5. Further immunohistochemistry stainings with CD31 showed that HMGA2 expression does not co-localize with blood vessels (Figure 8).

### Immunohistochemistry reveals HMGA2 expression in embryonic gingiva

HMGA2 nuclear immunoreactivity was abundant in cells of embryonic gingiva, in epithelial as well as in the mesenchymal compartments of 24.76 ± 9.57% (Additional file 6). Adult gingival tissue did not express discernible HMGA2 protein immunostaining (Additional file 7).

## Discussion

Humans are diphyodont and have two generations of teeth, where the second permanent set slowly begins to replace the first deciduous set around the age of six. Studies of molecular differences in pulpal cells from teeth extracted from children and adults, respectively, provide unique opportunities to compare general age-related differences in human stromal populations. The regenerative capacity of human tissue is much greater during early childhood compared to later stages in life [24]. We hypothesized that this would be reflected in age-related changes in molecular characteristics of human pulpal stromal cells.

For this purpose, we compared gene expression profiles of pulp cells from deciduous and permanent teeth using a microarray chip that is specifically designed for stem/progenitor cells. This was performed to evaluate their genetic signatures with regard to key differences related to self-renewal, proliferation and differentiation. Cells from deciduous teeth were obtained from children (3- to 12-years old), thus being younger than the permanent teeth cells we collected from adults (18- to 52-years old). For obvious reasons, there was no possibility to match deciduous and permanent teeth to the same donor, which is reflected in the microarray results (Figure 1A). However, with this taken into account, our results clearly show that there are differentially expressed genes in the deciduous and permanent teeth. Genes involved in cell division, mitosis, stemness and aging were differently expressed in favor of pulp cells from deciduous teeth. The results indicate a regeneration-favorable phenotype of pulp cells from deciduous teeth.

Somatic stem cells age with time, with a concomitant reduction in amounts, mitotic activity and self-renewable capacity [1,25,26]. This should be ascribed to age-dependent alterations in gene function. When mesenchymal cells from bone marrow were compared between adults and children, it was proposed that the bone marrow cells from the latter are more suitable for tissue engineering purposes because of age-related factors [3]. *HMGA2* is an example of a gene that confers self-renewal and proliferation capacity to stem cells. It is strongly expressed by neural stem cells/progenitors in the subventricular zone, but declines over time with age [27-29]. A recent study has shown that the senescence-related tumor suppressor gene *p16*^*Ink4a*^, which diminishes the self renewal capacity of cells and also reduces the expression of *HMGA2*, is increased in older permanent teeth compared to young deciduous teeth [30]. This agrees with our data, where *HMGA2* was differentially expressed with a substantial level in cells from deciduous teeth but not detected in cells from permanent pulps. An overall age-dependent decline in cellular expression of HMGA2 outside brain and teeth is suggested by our immunohistochemical observations in human gingiva, where embryonic tissue expressed widespread HMGA2 immunoreactivity while none was observed in adult samples.

It should be noted, that our results show that not all stromal cells in deciduous pulps are HMGA2 positive. Instead, HMGA2^+^ cells constitute a subpopulation of approximately 16% of the total number. The full phenotype/s of this subpopulation remains elusive, although the HMGA2 expression suggests that it may contain progenitor cells. Other investigators have demonstrated that stem/progenitor cells in teeth are associated with blood vessels [7,12]. We did not find that HMGA2-positive cells were related to blood vessels, which suggests that we might have found another proliferative subpopulation in deciduous teeth.

We attempted to clarify the role of HMGA2 in deciduous teeth pulp cells by reducing the level through siRNA transfections. Due to stable expression of *HMGA2* mRNA in the cells, we managed to suppress *HMGA2* levels to approximately 50% of those siControl untreated samples (Figure 4A). This resulted in a significant reduction of mRNA levels of *NANOG*, which is thought to be a key factor in maintaining pluripotency. This finding gives further indications that *HMGA2* has a key role in the regulation and maintenance of a subset of pulpal cells. *SOX2* levels were not detectable, which is expected since dental stem cells that express *SOX2* are epithelial and not mesenchymal. *OCT4* levels were not affected by the depletion of *HMGA2*.

Our results indicate that deciduous teeth harbor a population of cells, with a higher self-renewal and proliferation capacity than those of permanent teeth. Thus, markers involved in cell division and mitosis were up-regulated in deciduous cells compared to cells from permanent teeth (Table 2). This could make primary pulp cells more suitable for tissue regeneration. In fact, from certain aspects, they might even be more useful than BMMSCs, since the levels of stem cell markers NANOG and OCT4 were similar between these cell types, but HMGA2 was slightly higher in deciduous pulp cells (Figure 5A-C).

## Conclusions

Using specifically designed microarray panels, RT q-PCR, Western blot and immunohistochemistry we show that *HMGA2*, a stem cell-associated gene, is robustly expressed in deciduous pulp cells but not in permanent tooth pulp cells. Additionally, several genes associated with mitosis were highly expressed in deciduous pulp cells, while matrix genes and several signaling molecules were strongly expressed in the adult. These findings strongly suggest that there are molecular differences in cellular populations of different age and should serve as important basic information for understanding the therapeutic potential of pulpal cells. Specifically, primary pulp cells may be better suited for regenerative medicine and tissue engineering purposes than cells from permanent tooth pulps and based on this, are recommended for early banking.

## Additional files

Additional file 1:
**The 70 differently expressed genes in microarray,** ***P*** **≤0.05.**
Additional file 2:
**Network representation of** ***HMGA2*** **interacting genes in permanent compared to deciduous tooth.** Direct interacting, highly interconnected genes merged from Ingenuity Pathway analysis. Genes are represented as nodes and relations between genes, interactions, as lines. Gene modulation direction as a result of the comparison is color coded: green, down-regulated and red, up-regulated and color intensity represents strength of the modulation. Node shapes represent different gene classes. Additional file 3:
**Network representation of interacting genes in adult compared to deciduous tooth.** Top ranking networks of directly interacting, highly interconnected genes merged from Ingenuity Pathway analysis. Genes are represented as nodes and relations between genes, interactions, as lines. Gene modulation direction as a result of the comparison is color coded: green, down-regulated and red, up-regulated and color intensity represents strength of the modulation. Node shapes represent different gene classes. Genes are placed according to their subcellular localization. Additional file 4:
**Immunofluorescence intensity of HMGA2.** The immunofluorescence intensity was measured in HMGA2+ cells and negative control cells. Additional file 5:
**Hematoxylin & Eosin staining of deciduous and permanent tooth pulps.** The H&E staining shows the organization of tooth pulp. A) Deciduous tooth pulp, B) Permanent tooth pulp. Scale bar 200 μm. Additional file 6:
**Immunohistochemistry of HMGA2 expression in embryonic gingiva.** HMGA2 is abundantly expressed in epithelial and mesenchymal cells of embryonic gingiva, and is co-localized with nuclear DAPI staining. A) HMGA2 (green), CD31 (a blood vessel marker) (red) and DAPI (blue). B-E are zoomed in areas. .Scale bars. A 70 μm; B –E: 30 μm. Additional file 7:
**Immunohistochemistry of HMGA2 expression in adult gingiva.** HMGA2 expression is not observed in adult gingiva. A) HMGA2 (green), CD31 (a blood vessel marker) (red) and DAPI (blue). B-E are zoomed in areas. Scale bars: A 500 μm; B-C: 50 μm; D-E: 70 μm.

## Abbreviations

BMMSCs: bone marrow mesenchymal stem cells
HES: human embryonic stem cells
HMGA2: high-mobility group AT-hook 2
Ig: immunoglobulin
IPA: Ingenuity Pathway Analysis
OCT: optimum cutting temperature
PBS: phosphate-buffered saline
RT q-PCR: real time quantitative real-time polymerase chain reaction
siRNA: small interfering RNA
