# Supplementary material for: Molecular differences between stromal cell populations from deciduous and permanent human teeth
Source: Stem Cell Res Ther. 2015 Apr 18;6(1):59. doi: 10.1186/s13287-015-0056-7 (PMC4417277; doi:10.1186/s13287-015-0056-7)
Supplement: Additional file 1: — The 70 differently expressed genes in microarray, P ≤0.05. [file 13287_2015_56_MOESM1_ESM.doc]

**Supplemental table 1. All Differently Expressed Genes in MicroArray, p≤ 0.05**

| **Gene Name** | **Reference Sequence** | **Description** | **log_2_ fold change** | |
| --- | --- | --- | --- | --- |
| ABCC8 | NM_000352 | (ABCC8 OR SUR1 OR SUR) (ABC-TRANSPORTER) SULFONYLUREA RECEPTOR 1. | 0.215300899 | |
| ABCC9 | NM_005691 NM_020297 NM_020298 | (ABCC9 OR SUR2) ATP-BINDING CASSETTE TRANSPORTER SUB-FAMILY C MEMBER 9 (SULFONYLUREA RECEPTOR 2). | 0.224382829 | |
| AHNAK | NM_001620 | (AHNAK OR PM227) NEUROBLAST DIFFERENTIATION ASSOCIATED PROTEIN AHNAK (DESMOYOKIN) (FLJ33834). | 0.369845823 | |
| ALPL | NM_000478 | (ALPL) ALKALINE PHOSPHATASE, TISSUE-NONSPECIFIC ISOZYME PRECURSOR (EC 3.1.3.1) (AP-TNAP) (LIVER/BONE/KIDNEY ISOZYME) (TNSALP) (AKP2 OR AKP-2). | 0.593692642 | |
| BRCA1 | NM_007294 NM_007297 NM_007298 NM_007299 NM_007300 NR_027676 | (BRCA1 OR RNF53) BREAST CANCER TYPE 1 SUSCEPTIBILITY PROTEIN (RING FINGER PROTEIN 53). | -0.587936721 | |
| BUB1 | NM_004336 | (BUB1 OR BUB1L) MITOTIC CHECKPOINT SERINE/THREONINE-PROTEIN KINASE BUB1 (EC 2.7.1.-) (HBUB1) (BUB1A). | -0.810828215 | |
| BUB1B | NM_001211 | (BUB1B OR MAD3L OR BUBR1) MITOTIC CHECKPOINT SERINE/THREONINE-PROTEIN KINASE BUB1 BETA (EC 2.7.1.-) (HBUBR1) (MAD3/BUB1-RELATED PROTEIN KINASE) (MITOTIC CHECKPOINT KINASE MAD3L). | -0.836113829 | |
| BUB3 | NM_004725 | (BUB3) MITOTIC CHECKPOINT PROTEIN BUB3. | -0.43310528 | |
| CA14 | NM_012113 | (CA14) CARBONIC ANHYDRASE XIV PRECURSOR (EC 4.2.1.1) (CARBONATE DEHYDRATASE XIV) (CA-XIV). | -0.715784421 | |
| CCCAP | NM_006642 | (SDCCAG8 OR CCCAP) CENTROSOMAL COLON CANCER AUTOANTIGEN PROTEIN (HSPC085) (NY-CO-8) (2700048G21RIK) (5730470G24RIK) (SLINKY). | 0.249448005 | |
| CCNB2 | NM_004701 | (CCNB2) CYCLIN B2 G2/MITOTIC SPECIFIC CYCLIN B2. | -1.017401995 | |
| CDC25C | NM_022809 | (CDC25C) M-PHASE INDUCER PHOSPHATASE 3 (EC 3.1.3.48). | -0.502065811 | |
| CDC2A | NM_001786, NM_033379 | (CDC2 OR CDC2A) CELL DIVISION CONTROL PROTEIN 2 HOMOLOG (EC 2.7.1.22) (EC 2.7.1.23) (P34 PROTEIN KINASE) (CYCLIN-DEPENDENT KINASE 1) (CDK1). | -0.983460787 | |
| CDK4 | NM_000075 | (CDK4) CELL DIVISION PROTEIN KINASE 4 (EC 2.7.1.-) (CYCLIN-DEPENDENT KINASE4) (PSK-J3). | -0.394305598 | |
| CEBPA_3 | NM_004364 | (CEBPA) CCAAT/ENHANCER BINDING PROTEIN ALPHA (C/EBP ALPHA). | -0.539317108 | |
| COL1A1 | NM_000088 | (COL1A1) COLLAGEN ALPHA 1(I) CHAIN PRECURSOR. | 0.624552045 | |
| CTNNA1 | NM_001903 | (CTNNA1) ALPHA-1 CATENIN (CADHERIN-ASSOCIATED PROTEIN) (ALPHA E-CATENIN) (NY-REN-13 ANTIGEN). | 0.321948313 | |
| DTYMK | NM_012145 | (DTYMK OR TYMK OR TMPK OR CDC8) THYMIDYLATE KINASE (EC 2.7.4.9) (DTMP KINASE). | -0.779461232 | |
| EAAT1 | NM_004172 | (SLC1A3 OR EAAT1) EXCITATORY AMINO ACID TRANSPORTER 1 (SODIUM-DEPENDENT GLUTAMATE/ASPARTATE TRANSPORTER 1) (GLIAL GLUTAMATE TRANSPORTER) (GLAST1) | 0.300144029 | |
| FABE | NM_001444 | (FABP5 OR MAL1 OR KLBP OR FABPE) FATTY ACID-BINDING PROTEIN, EPIDERMAL (E-FABP) (PSORIASIS-ASSOCIATED FATTY ACID-BINDING PROTEIN HOMOLOG) (PA-FABP). | -0.995146092 | |
| FGFR1_1_HUMAN | NM_015850, NM_023105, NM_023106, NM_023107, NM_023108, NM_023110 | (FGFR1 OR FLG OR FGFBR OR FLT2) BASIC FIBROBLAST GROWTH FACTOR RECEPTOR 1 PRECURSOR (BFGF-R) EC 2.7.1.112) (FMS-LIKE TYROSINE KINASE-2) (C-FGR) (BFGFR) (CD331 ANTIGEN). | 0.234339081 | |
| FLJ21190 | NM_024096 | HYPOTHETICAL PROTEIN FLJ21190 (CDA03) (RS21C6) (TDRG-TL1 OR 2410015N17RIK) (RS21-C6). | -0.440062387 | |
| FLJ22362 | NM_022823 | (FNMP1) HYPOTHETICAL PROTEIN FLJ22362 (FRCP1 OR 2810430J06RIK). | 0.629864473 | |
| FN1 | NM_002026 NM_212474, NM_212475 NM_212476, NM_212478 NM_212482 | (FN1 OR FN) FIBRONECTIN PRECURSOR (FN) (COLD-INSOLUBLE GLOBULIN) (CIG). | 0.81389108 | |
| FN1_EIIIA | NM_002026 NM_212474, NM_212475 NM_212476, NM_212478 NM_212482 | (FN1 OR FN) FIBRONECTIN PRECURSOR (FIBRONECTIN EIIIA DOMAIN). | 0.7576541 | |
| **Differently Expressed Genes in MicroArray** | | | | |
| **Gene Name** | **Reference Sequence** | **Description** | **log_2_ fold** | |
| FN1_REPEAT-1TO6 | NM_002026 NM_212474, NM_212475 NM_212476, NM_212478 NM_212482 | (FN1 OR FN) FIBRONECTIN PRECURSOR (FN) (COLD-INSOLUBLE GLOBULIN) (CIG). | 0.664206958 | |
| FN1_REPEAT-A | NM_002026 NM_212474, NM_212475 NM_212476, NM_212478 NM_212482 | (FN1 OR FN) FIBRONECTIN PRECURSOR (FN) (COLD-INSOLUBLE GLOBULIN) (CIG). | 1.024595683 | |
| FN1_REPEAT-B | NM_002026 NM_212474, NM_212475 NM_212476, NM_212478 NM_212482 | (FN1 OR FN) FIBRONECTIN PRECURSOR (FN) (COLD-INSOLUBLE GLOBULIN) (CIG). | 1.050652139 | |
| FZD3 | NM_017412 | (FZD3) FRIZZLED 3 PRECURSOR (FRIZZLED-3) (FZ-3) (HFZ3) (MFZ3) (RFZ3). | -0.399842904 | |
| GGH | NM_003878 | (GGH) GAMMA-GLUTAMYL HYDROLASE PRECURSOR (EC 3.4.19.9) (GAMMA-GLU-X CARBOXYPEPTIDASE) (CONJUGASE) (GH). | -0.399654802 | |
| HIST1H2AC | NM_003512 | (HIST1H2AC OR H2AFL) HISTONE H2A.L (H2A/L). | 0.794069575 | |
| HMGIC | NM_003483 | (HMGA2 OR HMGIC) HIGH MOBILITY GROUP PROTEIN HMGI-C (HIGH MOBILITY GROUP AT-HOOK 2). | -1.390362456 | |
| HNRPA1 | NM_002136 NM_031157 | (HNRPA1) HETEROGENEOUS NUCLEAR RIBONUCLEOPROTEIN A1 (HELIX-DESTABILIZING PROTEIN) (SINGLE-STRAND BINDING PROTEIN) (HNRNP CORE PROTEIN A1). | -0.618882062 | |
| IGF1R | NM_000875 | (IGF1R) INSULIN-LIKE GROWTH FACTOR I RECEPTOR PRECURSOR (EC 2.7.1.112) (CD221 ANTIGEN). | 0.335180399 | |
| INHBA | NM_002192 | (INHBA) INHIBIN BETA A CHAIN PRECURSOR (ACTIVIN BETA-A CHAIN) (ERYTHROID DIFFERENTIATION PROTEIN) (EDF). | 1.203885023 | |
| ISL1 | NM_002202 | (ISL1) INSULIN GENE ENHANCER PROTEIN ISL-1 (ISLET-1). | 0.5621812 | |
| ITGB1 | NM_002211, NM_033666, NM_033667, NM_033668, NM_033669, NM_133376 | (ITGB1 OR FNRB) INTEGRIN BETA-1 PRECURSOR (FIBRONECTIN RECEPTOR BETA SUBUNIT) (CD29 ANTIGEN) (INTEGRIN VLA-4 BETA SUBUNIT). | 0.446738627 | |
| JMJ | NM_004973 | (JMJ) JUMONJI PROTEIN. | 0.237936732 | |
| JUNB | NM_002229 | (JUNB) TRANSCRIPTION FACTOR JUN-B (G0S3). | 0.540422583 | |
| KCNA1 | NM_000217 | (KCNA1) VOLTAGE-GATED POTASSIUM CHANNEL PROTEIN KV1.1 (HUKI) (HBK1). | 0.305040488 | |
| KPNA2 | NM_002266 | (KPNA2 OR RCH1 OR SRP1) IMPORTIN ALPHA-2 SUBUNIT (KARYOPHERIN ALPHA-2 SUBUNIT) (SRP1-ALPHA) (RAG COHORT PROTEIN 1). | -1.129768742 | |
| LAMA3 | NM_000227, NM_198129 | (LAMA3) LAMININ ALPHA-3 CHAIN PRECURSOR (EPILIGRIN 170 KDA SUBUNIT) (E170). | 0.440951984 | |
| LAMG1 | NM_002293 | (LAMC1 OR LAMB2) LAMININ GAMMA-1 CHAIN PRECURSOR (LAMININ B2 CHAIN). | 0.831512521 | |
| MAD2L1 | NM_002358 | (MAD2L1 OR MAD2 OR MAD2A) MITOTIC SPINDLE ASSEMBLY CHECKPOINT PROTEIN MAD2A (MAD2-LIKE 1). | -0.970346213 | |
| MGST1 | NM_020300 NM_145764 NM_145791 NM_145792 | (MGST1 OR MGST OR GST12) GLUTATHIONE S-TRANSFERASE, MICROSOMAL (EC 2.5.1.18). | 0.825014458 | |
| MMP2 | NM_004530 | (MMP2 OR CLG4A) 72 KDA TYPE IV COLLAGENASE PRECURSOR (EC 3.4.24.24) (72 KDA GELATINASE) (MATRIX METALLOPROTEINASE-2) (MMP-2) (GELATINASE A) (TBE-1) | 1.047277143 | |
| MMP23A-MMP23B | NM_006983 NR_002946 | ((MMP23A OR MMP21) AND (MMP23B OR MMP21 OR MMP22)) MATRIX METALLOPROTEINASE-23 PRECURSOR (EC 3.4.24.-) (MMP-23) (MATRIX METALLOPEPTIDASE 21) (MMP-21) (MATRIX METALLOPROTEASE 22) (MMP-22) (FEMALYSIN) (MIFR-1) | -0.485888989 | |
| MMP6 | NM_005792 | (MPHOSPH6 OR MPP6) M-PHASE PHOSPHOPROTEIN 6. | -0.377538409 | |
| MYL4 | NM_002476 | (MYL4 OR MLC1) MYOSIN LIGHT CHAIN 1, EMBRYONIC MUSCLE/ATRIAL ISOFORM (PRO1957). MYOSIN LIGHT CHAIN ALKALI, GT-1 ISOFORM (FRAGMENT). | 0.442391978 | |
| **Differently Expressed Genes in MicroArray** | | | | |
| **Gene Name** | **Reference Sequence** | **Description** | **log_2_ fold** | |
| NOP5 | NM_015934 | (NOP5) NUCLEOLAR PROTEIN NOP5 (NUCLEOLAR PROTEIN 5) (NOP58) (HSPC120) (NOL5) (SIK SIMILAR PROTEIN). | -0.501750824 | |
| NPM1 | NM_001004419, NM_002520, NM_013269, NM_199185 | (NPM1 OR NPM) NUCLEOPHOSMIN (NPM) (NUCLEOLAR PHOSPHOPROTEIN B23) (NUMATRIN) (NUCLEOLAR PROTEIN NO38). | -0.703825099 | |
| NPPA | NM_006172 | (NPPA OR PND) ATRIAL NATRIURETIC FACTOR PRECURSOR (ANF) (ATRIAL NATRIURETIC PEPTIDE) (ANP) (PREPRONATRIODILATIN). | -0.41397212 | |
| PDGFB | NM_002608 NM_033016 | (PDGFB OR PDGF2 OR SIS) PLATELET-DERIVED GROWTH FACTOR B CHAIN PRECURSOR (PDGF B-CHAIN) (PLATELET-DERIVED GROWTH FACTOR BETA POLYPEPTIDE) (PDGF-2) (C-SIS) (BECAPLERMIN). | 0.486227152 | |
| PITX2 | NM_000325 NM_153426 NM_153427 | (PITX2 OR RIEG1 OR RIEG OR RGS OR ARP1) PITUITARY HOMEOBOX 2 (RIEG BICOID-RELATED HOMEOBOX TRANSCRIPTION FACTOR) (SOLURSHIN) (ALL1 RESPONSIVE PROTEIN ARP1). | -0.239366141 | |
| PLXNA3 | NM_017514 | (PLXNA3 OR PLXN4 OR SEX) PLEXIN A3 PRECURSOR (PLEXIN 4) (TRANSMEMBRANE PROTEIN SEX) (PLXN3) (PLEXIN 3). | 0.293640907 | |
| PPP2R1B_2 | NM_181699 | (PPP2R1B) SERINE/THREONINE PROTEIN PHOSPHATASE 2A, 65 KDA REGULATORY SUBUNIT A,BETA ISOFORM (PP2A, SUBUNIT A, PR65-BETA ISOFORM) (PP2A, SUBUNIT A,R1-BETA ISOFORM) (TRANSCRIPT VARIANT 2). | 0.369964398 | |
| PRKCM | NM_002742 | (PRKCM) PROTEIN KINASE C, MU TYPE (EC 2.7.1.-) (NPKC-MU). | 0.425164376 | |
| PTTG_HUMAN | NM_004219, NM_006607 NR_002734 | ((PTTG1 OR EAP1 OR PTTG OR TUTR1) AND (PTTG2) AND (PTTG3)) SECURIN (PITUITARY TUMOR-TRANSFORMING PROTEIN 1) (TUMOR-TRANSFORMING PROTEIN 1) (ESP1-ASSOCIATED PROTEIN) (HPTTG) (PITUITARY TUMOR TRANSFORMING GENE PROTEIN 2) (PITUITARY TUMOR-TRANSFO | -1.116178205 | |
| RAMP1 | NM_005855 | (RAMP1) RECEPTOR ACTIVITY MODIFYING PROTEIN 1. | 0.874730335 | |
| RNASE4 | NM_002937 | (RNASE4 OR RNS4) RIBONUCLEASE 4 PRECURSOR (EC 3.1.27.-) (RNASE 4). | 0.642428919 | |
| SERPINF1 | NM_002615 | (SERPINF1 OR PEDF OR SDF3) PIGMENT EPITHELIUM-DERIVED FACTOR PRECURSOR (PEDF) (EPC-1) (STROMAL CELL-DERIVED FACTOR 3) (SDF-3) (CASPIN). | 0.897305456 | |
| SNRPF | NM_003095 | (SNRPF OR PBSCF) SMALL NUCLEAR RIBONUCLEOPROTEIN F (SNRNP-F) (SM PROTEIN F) (SM-F) (SMF). | -0.480652881 | |
| TIMP1 | NM_003254 | (TIMP1 OR TIMP OR CLGI) METALLOPROTEINASE INHIBITOR 1 PRECURSOR (TIMP-1) (ERYTHROID POTENTIATING ACTIVITY) (EPA) (TISSUE INHIBITOR OF METALLOPROTEINASES) (FIBROBLAST COLLAGENASE INHIBITOR) (COLLAGENASE INHIBITOR). | 0.603966738 | |
| TK1 | NM_003258 | (TK1) THYMIDINE KINASE, CYTOSOLIC (EC 2.7.1.21). | -0.910206932 | |
| TTR | NM_000371 | (TTR OR PALB) TRANSTHYRETIN PRECURSOR (PREALBUMIN) (TBPA) (TTR) (ATTR). | 0.365004639 | |
| TUBA_HUMAN | NM_006009 NM_006082 NM_032704 | ((TUBA1B) AND (TUBA1A) AND (TUBA1C)) TUBULIN ALPHA-UBIQUITOUS CHAIN (ALPHA-TUBULIN UBIQUITOUS) (TUBULIN K-ALPHA-1) (TUBA6) (TUBULIN ALPHA-6 CHAIN) (ALPHA-TUBULIN 6) (TUBA3) (TUBULIN ALPHA-3 CHAIN) (ALPHA-TUBULIN 3) (TUBULIN B-ALPHA-1). | -0.463266659 | |
| UBE2T | NM_014176 | (UBE2T OR HSPC150) UBIQUITIN-CONJUGATING ENZYME E2 T (EC 6.3.2.19) (UBIQUITIN-PROTEIN LIGASE T) (UBIQUITIN CARRIER PROTEIN T) (FLJ20497) (2700084L22RIK). | -0.941007535 | |
| VEGF | NM_001025366, NM_001025367, NM_001025368, NM_001025369 | (VEGF OR VEGFA) VASCULAR ENDOTHELIAL GROWTH FACTOR PRECURSOR (VEGF-A) (VASCULAR PERMEABILITY FACTOR) (VPF). | 0.35783097 |  |
| VWF | NM_000552 | (F8VWF OR VWF) VON WILLEBRAND FACTOR PRECURSOR. | 0.269424353 |  |
| WISP3 | NM_003880 NM_198239 | (WISP3 OR CCN6 OR DJ142L7.3 OR LIBC) WNT1 INDUCIBLE SIGNALING PATHWAY PROTEIN 3 PRECURSOR (WISP-3) (CONNECTIVE TISSUE GROWTH FACTOR (NOV, GIG) LIKE PROTEIN (WISP3) (CONNECTIVE TISSUE GROWTH FACTOR RELATED PROTEIN WISP-3) | 0.16039008 |  |
