# Supplementary material for: Molecular differences between stromal cell populations from deciduous and permanent human teeth
Source: Stem Cell Res Ther. 2015 Apr 18;6(1):59. doi: 10.1186/s13287-015-0056-7 (PMC4417277; doi:10.1186/s13287-015-0056-7)
Supplement: Additional file 2: — Network representation of HMGA2 interacting genes in permanent compared to deciduous tooth. Direct interacting, highly interconnected genes merged from Ingenuity Pathway analysis. Genes are represented as nodes and relations between genes, interactions, as lines. Gene modulation direction as a result of the comparison is color coded: green, down-regulated and red, up-regulated and color intensity represents strength of the modulation. Node shapes represent different gene classes. [file 13287_2015_56_MOESM2_ESM.pdf]

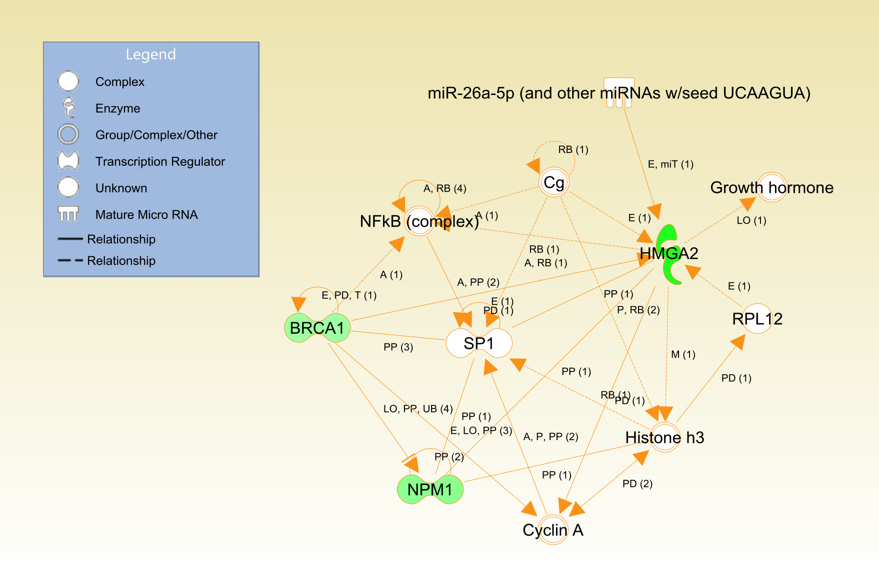

Legend
Complex
Enzyme
Group/Complex/Other
Transcription Regulator
Unknown
Mature Micro RNA
Relationship
Relationship
miR-26a-5p (and other miRNAs w/seed UCAAGUA)
E, miT (1)
RB (1)
Cg
Growth hormone
LO (1)
A, RB (4)
NFkB (complex)
A (1)
E (1)
HMGA2
RB (1)
A, RB (1)
A (1)
A, PP (2)
E, PD, T (1)
E (1)
PD (1)
PP (1)
P, RB (2)
E (1)
RPL12
BRCA1
SP1
PP (3)
M (1)
PD (1)
PP (1)
RB (1)
PD (1)
LO, PP, UB (4)
PP (1)
E, LO, PP (3)
A, P, PP (2)
Histone h3
PP (2)
NPM1
PP (1)
PD (2)
Cyclin A
