# Supplementary figures and images for: Molecular differences between stromal cell populations from deciduous and permanent human teeth
Source: Stem Cell Res Ther. 2015 Apr 18;6(1):59. doi: 10.1186/s13287-015-0056-7 (PMC4417277; doi:10.1186/s13287-015-0056-7)

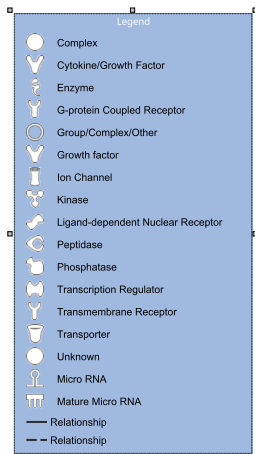

## Extracellular Space

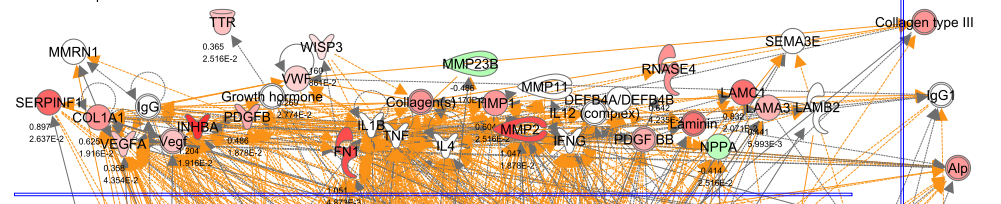

## Plasma Membrane

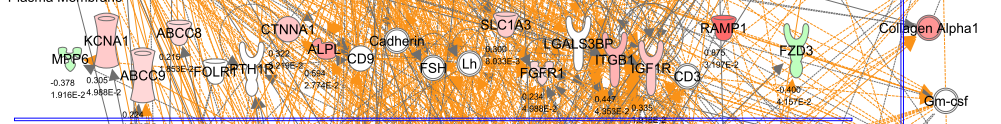

## Cytoplasm

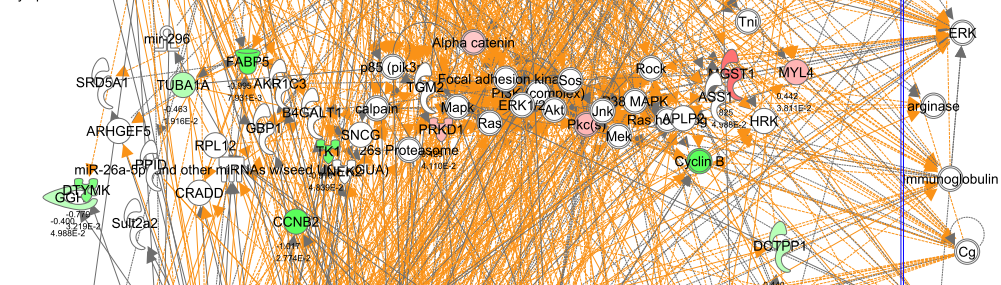

## Nucleus

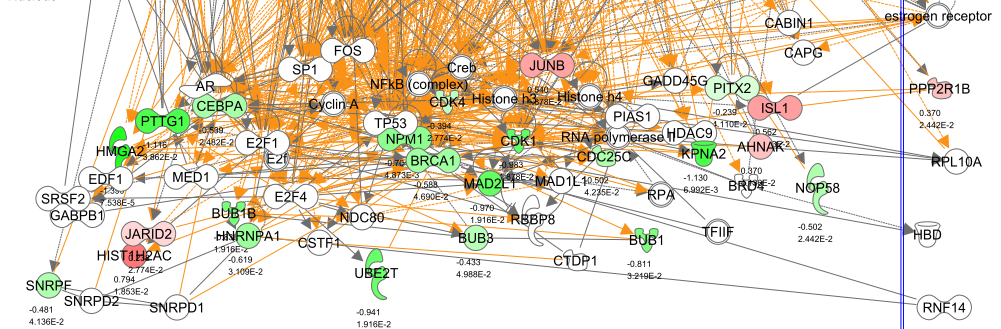

Supplement: Additional file 3: — Network representation of interacting genes in adult compared to deciduous tooth. Top ranking networks of directly interacting, highly interconnected genes merged from Ingenuity Pathway analysis. Genes are represented as nodes and relations between genes, interactions, as lines. Gene modulation direction as a result of the comparison is color coded: green, down-regulated and red, up-regulated and color intensity represents strength of the modulation. Node shapes represent different gene classes. Genes are placed according to their subcellular localization. [file 13287_2015_56_MOESM3_ESM.pdf]
